# Changes in posterior corneal elevations after combined transepithelial photorefractive keratectomy and accelerated corneal collagen cross-linking: retrospective, comparative observational case series

**DOI:** 10.1186/s12886-016-0320-3

**Published:** 2016-08-08

**Authors:** Hun Lee, David Sung Yong Kang, Byoung Jin Ha, Jin Young Choi, Eung Kweon Kim, Kyoung Yul Seo, Tae-im Kim

**Affiliations:** 1 Department of Ophthalmology, International St. Mary’s Hospital, Catholic Kwandong University College of Medicine, Incheon, South Korea; 2 The Institute of Vision Research, Department of Ophthalmology, Yonsei University College of Medicine, Seoul, South Korea; 3 Eyereum Eye Clinic, Seoul, South Korea; 4 Corneal Dystrophy Research Institute, Department of Ophthalmology, Yonsei University College of Medicine, Seoul, South Korea

**Keywords:** Posterior corneal elevations, Transepithelial photorefractive keratectomy, Accelerated corneal collagen cross-linking, Scheimpflug tomography, Best-fit toric ellipsoid

## Abstract

**Background:**

To compare the changes in anterior and posterior corneal elevations after combined transepithelial photorefractive keratectomy (PRK) and accelerated corneal collagen cross-linking (CXL) and after PRK.

**Methods:**

Medical records of 82 eyes of 44 patients undergoing either combined transepithelial PRK and CXL (PRK-CXL group) or transepithelial PRK (PRK group) were examined retrospectively. Changes in anterior and posterior corneal elevations were calculated by fitting an 8.0-mm diameter best-fit sphere and best-fit toric ellipsoid (BFTE) to the corneal shape with a fixed eccentricity of 0.4 using Scheimpflug tomography (Pentacam HR; Oculus Optikgeräte GmbH, Wetzlar, Germany) preoperatively and 6 months postoperatively.

**Results:**

In anterior corneal elevation, both groups demonstrated a similar trend of a forward displacement of peripheral anterior corneal surface and a backward displacement of central anterior corneal surface. In posterior corneal elevation, a forward displacement of peripheral posterior corneal surface was induced in both groups, along with a backward displacement of central posterior corneal surface, regardless of the calculation method. The magnitudes of displacement of peripheral and central posterior corneal surfaces were significantly smaller in the PRK-CXL group than in the PRK group. Moreover, the PRK-CXL group showed a backward displacement of posterior corneal surface at maximum corneal elevations when the BFTE was used as the reference surface.

**Conclusions:**

Transepithelial PRK combined with prophylactic CXL significantly reduced the magnitudes of displacement of peripheral and central posterior corneal surfaces, with the radius of the BFTE was set to 8.0-mm on the Scheimpflug tomography system.

## Background

Elevation of the posterior corneal surface can occur after myopic photorefractive keratectomy (PRK) and laser in-situ keratomileusis (LASIK) [1–3]. Early studies using scanning-slit topography (Orbscan; Bausch & Lomb, Rochester, NY, USA) showed forward displacement of the posterior corneal surface, whereas later studies using the Scheimpflug tomography system (Pentacam HR; Oculus Optikgeräte GmbH, Wetzlar, Germany) showed minimal or no changes in posterior corneal elevations [1, 3–6]. In addition, differences in methods of measurement of posterior corneal elevation could considerably affect the results [6–10]. Accurate and reliable measurement of posterior corneal elevations is crucial because posterior corneal elevation after myopic laser refractive surgery could indicate postoperative ectasia.

Collagen cross-linking (CXL) is a recently developed surgical procedure whereby riboflavin sensitization with ultraviolet-A (UVA) radiation induces stromal cross-linking [11]. This procedure alters corneal biomechanics and increases mechanical rigidity (i.e., strengthens the corneal tissue) in porcine and human corneas, resulting in significant increases in the stiffness of the anterior corneal stroma [12]. Clinically, patients with keratoconus, ectasia following photorefractive surgery, and even corneal infections and chemical burns can benefit from CXL [13, 14]. A recently introduced accelerated CXL protocol consisting of higher-intensity light applied for a shorter period of time can be applied in various clinical settings [15]. The outcomes of this protocol are comparable to those of conventional CXL, with no evidence of endothelial cell density changes [16].

Application of prophylactic CXL concurrently with myopic LASIK surgery may increase corneal stabilization following photorefractive surgery. Several studies have evaluated the safety and efficacy of combined LASIK and CXL, reporting no signs of ectasia or significant regression during the follow-up period [17, 18]. Furthermore, concurrent CXL and myopic LASIK surgery improves refractive and keratometric stability to a greater degree than does standard LASIK alone [19].

The Scheimpflug tomography system evaluates corneal indices by providing multiple corneal descriptors. Additionally, it allows measurements of local elevation points by fitting the corneal shape to a best-fit sphere (BFS) or best-fit toric ellipsoid (BFTE) reference surface. Scheimpflug tomography reveals posterior corneal elevation values by analyzing the posterior corneal surface directly [4, 5, 8]. Moreover, posterior corneal elevation measurements obtained with Scheimpflug tomography have relatively acceptable reproducibility and repeatability in both normal eyes and eyes with keratoconus and a history of CXL [10, 20, 21].

Because of the positive effects of concurrent prophylactic CXL, we hypothesized that combined application of prophylactic CXL and transepithelial PRK would have a positive effect on refractive outcomes and posterior corneal elevations. To the best of our knowledge, no studies have evaluated the effects of combined transepithelial PRK and accelerated corneal CXL on changes in anterior and posterior corneal elevations. Therefore, we aimed to investigate changes in anterior and posterior corneal elevations using Scheimpflug tomography after combined transepithelial PRK and accelerated corneal CXL and after transepithelial PRK alone.

## Methods

We performed this retrospective, comparative observational case series with the approval of the Institutional Review Board of Yonsei University College of Medicine (Seoul, South Korea). All study conduct adhered to the tenets of the Declaration of Helsinki and followed good clinical practices. All patients provided written informed consent for their medical information to be included in study analyses. Patients included in the study were older than 20 years and underwent either combined transepithelial PRK and accelerated corneal CXL (PRK-CXL group) or transepithelial PRK alone (PRK group) in a standardized fashion by the same surgeon between August 2014 and March 2015. We excluded patients with previous ocular or intraocular surgery, ocular abnormalities other than myopia or myopic astigmatism with a corrected distance visual acuity (CDVA) of 1.00 (20/20 Snellen) or better in both eyes, central corneal thickness (CCT) of less than 460 μm, corneal endothelial cell density of less than 2000 cells/mm^2^, cataract, ocular inflammation, or infection. We also excluded patients with signs of keratoconus on Scheimpflug tomography (displacement of the corneal apex, decrease in thinnest-point pachymetry, and asymmetric topographic pattern). According to our study protocol, we used combined transepithelial PRK and accelerated corneal CXL if a patient had any of the following preoperative measurements: CCT less than 500 μm regardless of the amount of ablation or predicted residual postoperative stromal thickness less than 300 μm. We retrospectively reviewed the medical records of 44 patients (82 eyes) meeting the inclusion and exclusion criteria.

### Examinations and measurements

Before and 6 months after surgery, all patients underwent complete ophthalmic examinations that included examinations for uncorrected distance visual acuity (UDVA) and CDVA, manifest refraction, slit-lamp examination (Haag-Streit, Gartenstadtstrasse, Köniz, Switzerland), intraocular pressure measurement (noncontact tonometer; NT-530, NCT Nidek Co., Ltd., Aichi, Japan), and fundus examination. Keratometric values and CCT were measured using autokeratometry (ARK-530A; Nidek Co., Ltd.) and ultrasound pachymetry (UP-1000; Nidek Co., Ltd.), respectively.

We measured the anterior and posterior corneal elevations without dilation preoperatively and at 6 months postoperatively using Scheimpflug tomography (Pentacam HR). We analyzed anterior and posterior corneal elevations at the peripheral (including 6 mm and 4 mm) and central (including 2 mm and center) zones by using an 8.0-mm-diameter area to calculate the BFS and BFTE fixed to the corneal apex defined by Scheimpflug tomography with a fixed eccentricity of 0.4. We also analyzed anterior and posterior maximum corneal elevations over the entire cornea. We determined changes in corneal elevations by subtracting the preoperative elevation from the postoperative elevation. The same reference surfaces were used to compare preoperative and postoperative states for direct comparison purposes. Forward protrusion of the anterior and posterior corneal surface resulted in a positive number. The same investigator performed all measurements, and we analyzed only high-quality measurements (quality score ≥90 %). The investigator performed each measurement 3 times, and we analyzed the average of the 3 measurements.

### Surgical technique

Transepithelial PRK was performed using an excimer laser (Amaris 1050 Excimer Laser platform; Schwind eye-tech-solutions GmbH and Co KG, Kleinostheim, Germany). After excimer laser ablation was complete, patients in the PRK-CXL group were treated with 0.1 % riboflavin with hydroxypropyl methylcellulose (Vibex Rapid; Avedro Inc, Waltham, MA, USA) placed on the corneal surface and carefully spread with an irrigating cannula for 120 s. After 120 s of soaking time, the corneal surface was rinsed thoroughly with 60 cm^3^ of chilled balanced salt solution (BSS). A UVA beam (wavelength, 365 nm) 9.0 mm in diameter was applied to the cornea in a pulsatile fashion (1:1) in a uniform circular pattern by the KXL system (Avedro Inc). The UVA exposure was performed for 180 s at a power of 30 mW/cm^2^ (total dose, 2.7 J/cm^2^). Mitomycin 0.02 % was applied to all corneas for 20 s after cessation of UVA irradiation, followed by thorough rinsing with chilled BSS.

Postoperatively, 1 drop of topical levofloxacin 0.5 % (Cravit; Santen Pharmaceutical, Osaka, Japan) was instilled at the surgical site, and a bandage contact lens (Acuvue Oasys; Johnson & Johnson Vision Care, Inc, Jacksonville, FL, USA) was placed on the cornea for both groups. Following surgery, topical levofloxacin 0.5 % and fluorometholone 0.1 % (Flumetholon; Santen Pharmaceutical) were applied 4 times per day for 1 month. The dosage was tapered over 3 months.

### Statistical analysis

Statistical analysis was performed using SAS software (version 9.2; SAS Institute, Inc, Cary, NC, USA). Results are expressed as mean ± standard deviation. We used the Kolmogorov–Smirnov test to confirm data normality. To statistically compare data from eyes that underwent combined transepithelial PRK and CXL or transepithelial PRK alone, we used independent *t* tests for continuous variables and *χ*^2^ tests for categorical variables. We performed paired *t* tests to evaluate the differences between preoperative and 6-month postoperative corneal elevation values in each group. We used independent *t* tests to compare changes in corneal elevation values between eyes that underwent combined transepithelial PRK and CXL and those that underwent transepithelial PRK alone. We used Pearson correlation analysis to evaluate the correlation between changes in posterior corneal elevation and preoperative parameters, including age, optical zone diameter, total ablation zone diameter, ablation depth, mean keratometric values, CCT, and spherical equivalent, in the PRK-CXL and PRK groups. In addition, we performed multiple regression analysis to evaluate the impact of preoperative parameters on changes in corneal elevation in the PRK-CXL and PRK groups. A *P* value of less than 0.05 was considered statistically significant.

## Result

This study included 82 eyes of 44 patients (18 women, 26 men). The mean patient age was 25.4 ± 5.1 years (range, 20 to 38 years). The analysis revealed that there was no significant difference between the PRK-CXL and PRK groups in age, spherical or cylindrical refractive error, spherical equivalent refraction, keratometric values, optical zone, total ablation zone, or ablation depth, except for CCT (Table 1).

**Table 1:** Characteristics of eyes that underwent combined transepithelial photorefractive keratectomy and accelerated corneal collagen cross-linking and transepithelial photorefractive keratectomy alone

Characteristics	PRK-CXL group (*n =* 40)	PRK group (*n =* 42)	*P*
Age, years old	25.8 ± 5.7 (20 to 38)	25.1 ± 4.6 (20 to 36)	.542
Sex (% women)	43 %	38 %	.717
Refractive errors (D)			
Spherical	–5.46 ± 1.20 (–7.50 to–3.00)	–5.78 ± 1.22 (–8.00 to–2.62)	.233
Cylindrical	–1.44 ± 0.73 (–2.62 to 0)	–1.27 ± 0.83 (–3.25 to 0)	.321
Spherical equivalent	–6.18 ± 1.28 (–8.12 to–3.50)	–6.42 ± 1.17 (–8.75 to–3.81)	.385
Keratometric value			
Flat K	42.3 ± 1.1 (40.3 to 44.3)	42.6 ± 1.3 (39.3 to 44.8)	.242
Steep K	44.1 ± 1.2 (41.0 to 46.0)	44.2 ± 1.5 (41.0 to 47.0)	.748
Preoperative CDVA	1.00 ± 0.02 (1.00 to 1.00)	1.01 ± 0.04 (1.00 to 1.00)	.160
Preoperative UDVA	0.04 ± 0.03 (0.01 to 0.15)	0.04 ± 0.02 (0.02 to 0.10)	.755
Optical zone (mm)	6.36 ± 0.23 (5.80 to 6.90)	6.32 ± 0.16 (6.00 to 6.70)	.280
Total ablation zone (mm)	8.10 ± 0.28 (7.45 to 8.64)	8.17 ± 0.36 (7.66 to 9.00)	.335
Ablation depth (μM)	101.54 ± 19.65 (54.13 to 128.68)	97.71 ± 20.56 (48.54 to 146.94)	.391
CCT (μM)	510.2 ± 32.9 (460.0 to 590.0)	537.8 ± 23.2 (501.0 to 610.0)	<.001

Results are expressed as means ± standard deviation (range)

PRK-CXL = Combined transepithelial photorefractive keratectomy and accelerated corneal collagen cross-linking; PRK = transepithelial photorefractive keratectomy; D = diopters; K = keratometric value; CDVA = corrected distance visual acuity; UDVA = uncorrected distance visual acuity; CCT = central corneal thickness

In the PRK-CXL group, the mean UDVA was 0.04 ± 0.03 preoperatively and 1.22 ± 0.25 at 6 months postoperatively (*P* < 0.001). The mean manifest refraction spherical equivalent (MRSE) was −6.18 ± 1.28 diopters (D) preoperatively and 0.03 ± 0.53D at 6 months postoperatively (*P* < 0.001). In the PRK group, mean UDVA was 0.04 ± 0.02 preoperatively and 1.27 ± 0.22 at 6 months postoperatively (*P* < 0.001). The mean MRSE was −6.42 ± 1.17D preoperatively and −0.04 ± 0.65D at 6 months postoperatively (*P* < 0.001).

Table 2 demonstrates the results of the comparison of anterior corneal elevation values between the PRK-CXL and PRK groups by fitting the corneal shape to the BFS or BFTE reference surface. Both groups showed significant forward displacement of the peripheral anterior corneal surface (including the 6-mm and 4-mm zones) with both the BFS and BFTE (*P* < 0.001). In cases with central anterior corneal elevations, backward displacements of the central anterior corneal surface (including the 2-mm zone and center) were statistically significant in both groups with both the BFS and BFTE (*P* < 0.001).

**Table 2:** Comparison of anterior corneal elevation values by fitting the corneal shape to a best-fit sphere or best-fit toric ellipsoid reference surface between eyes that underwent combined transepithelial photorefractive keratectomy and accelerated corneal collagen cross-linking and transepithelial photorefractive keratectomy alone

	PRK-CXL group (*n =* 40)				PRK group (*n =* 42)				*P* ^b^
	Pre	6 mon	Δ	*P* ^a^	Pre	6 mon	Δ	*P* ^a^	
Peripheral zone									
Ant. elevation (μm, BFS)	–3.59±1.04	9.82±6.76	13.41±6.42	<.001	–3.35±1.17	11.55±5.21	14.90±4.83	<.001	.123
Ant. elevation (μm, BFTE)	–0.27±1.17	12.27±5.32	12.54±6.42	<.001	–0.20±1.13	11.78±5.33	11.98±5.07	<.001	.524
Central zone									
Ant. elevation (μm, BFS)	4.38±1.62	–9.19±7.33	–13.57±7.26	<.001	4.08±1.65	–10.36±5.95	–14.44±5.87	<.001	.442
Ant. elevation (μm, BFTE)	0.78±1.75	–10.85±6.76	–11.63±6.60	<.001	0.79±1.52	–10.86±6.64	–11.65±6.57	<.001	.988
Max. elevation									
Ant. elevation (μm, BFS)	13.29±5.39	20.23±6.74	6.94±7.23	<.001	9.36±4.72	17.34±5.12	7.98±5.05	<.001	.320
Ant. elevation (μm, BFTE)	2.78±4.13	13.73±4.05	10.95±5.54	<.001	2.43±1.27	12.79±4.03	10.36±4.22	<.001	.500

PRK-CXL = Combined transepithelial photorefractive keratectomy and accelerated corneal collagen cross-linking; PRK = transepithelial photorefractive keratectomy; Δ = change; Peripheral zone = measurements of 6-mm and 4-mm zone; BFS = best-fit sphere; BFTE = best-fit toric ellipsoid; Central zone = measurements of 2-mm zone and center; Max. elevation = measurements of maximal elevation zone

^a^ *P* value between preoperative and 6-month postoperative corneal elevation values in each group

^b^ *P* value between changes in preoperative and postoperative corneal elevation values of PRK-CXL and PRK group

**Fig. 1:**
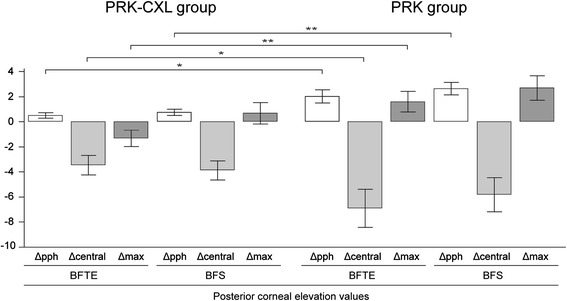
Comparison of changes in preoperative and postoperative corneal elevation values between eyes that underwent combined transepithelial photorefractive keratectomy and accelerated corneal collagen cross-linking and transepithelial photorefractive keratectomy alone. PRK-CXL = Combined transepithelial photorefractive keratectomy and accelerated corneal collagen cross-linking; PRK = transepithelial photorefractive keratectomy; Δ = change; pph = measurements of 6-mm and 4-mm zone; central = measurements of 2-mm zone and center; max = measurements of maximal elevation zone; BFTE = best-fit toric ellipsoid; BFS = best-fit sphere. Error bars represent standard error of the mean (*p < 0.05, **p < 0.01)

Table 3 demonstrates the results of the comparison between the two groups in posterior corneal elevation values by fitting the corneal shape to the BFS or BFTE reference surface. Both groups showed significant forward displacement of the peripheral posterior corneal surface with both the BFS and BFTE. However, there were statistically significant differences in posterior corneal elevation changes between the two groups (*P* = 0.001 for the BFS and *P* = 0.010 for the BFTE), although the magnitudes were small (<2 μm; Fig. 1).

**Table 3:** Comparison of posterior corneal elevation values by fitting the corneal shape to a best-fit sphere or best-fit toric ellipsoid reference surface between eyes that underwent combined transepithelial photorefractive keratectomy and accelerated corneal collagen cross-linking and transepithelial photorefractive keratectomy alone

	PRK-CXL group (*n =* 40)				PRK group (*n =* 42)				*P* ^b^
	Pre	6 mon	Δ	*P* ^a^	Pre	6 mon	Δ	*P* ^a^	
Peripheral zone									
Post. elevation (μm, BFS)	–4.46 ± 4.37	–3.72 ± 4.58	0.75±1.58	.005	–3.79±4.46	–1.16±4.99	2.63±3.24	<.001	.001
Post. elevation (μm, BFTE)	–0.38 ± 4.45	0.12±4.40	0.50±1.35	.024	0.59±4.44	2.61±5.22	2.02±3.43	<.001	.010
Central zone									
Post. elevation (μm, BFS)	4.11±5.80	0.23±5.46	–3.88 ± 4.84	<.001	3.30±7.28	–2.52 ± 7.01	–5.82 ± 8.81	<.001	.220
Post. elevation (μm, BFTE)	1.31 ± 6.67	–2.16 ± 5.32	–3.47 ± 4.86	<.001	–1.26±7.33	–8.17 ± 7.59	–6.90 ± 9.90	<.001	.049
Max. elevation									
Post. elevation (μm, BFS)	20.28 ± 7.18	20.95 ± 7.80	0.68±5.34	.429	19.5±7.19	22.26 ± 9.64	2.69±6.35	.009	.124
Post. elevation (μm, BFTE)	9.10±3.36	7.78±4.00	–1.33±4.08	.047	9.29±3.93	10.88±3.51	1.60±5.31	.059	.007

PRK-CXL = Combined transepithelial photorefractive keratectomy and accelerated corneal collagen cross-linking; PRK = transepithelial photorefractive keratectomy; Δ = change; Peripheral zone = measurements of 6-mm and 4-mm zone; BFS = best-fit sphere; BFTE = best-fit toric ellipsoid; Central zone = measurements of 2-mm zone and center; Max. elevation = measurements of maximal elevation zone

^a^ *P* value between preoperative and 6-month postoperative corneal elevation values in each group

^b^ *P* value between changes in preoperative and postoperative corneal elevation values of PRK-CXL and PRK group

Both groups showed significant backward displacement of the central posterior corneal surface with both the BFS and BFTE (*P* < 0.001; Table 3). However, compared with the PRK group, the PRK-CXL group showed a significantly small magnitude of backward displacement of the central posterior corneal surface when calculated with the BFTE (*P* = 0.049; Fig. 1).

When analyzing posterior maximum corneal elevations, the PRK group showed a significant forward displacement of the posterior corneal surface with the BFS (*P* = 0.009). When calculated with the BFTE, the PRK group showed forward displacement of the posterior corneal surface, although it did not reach statistical significance. The PRK-CXL group showed no significant change when calculated with the BFS and showed a significant backward displacement of the posterior corneal surface when calculated with the BFTE (*P* = 0.047; Fig. 1).

**Fig. 2:**
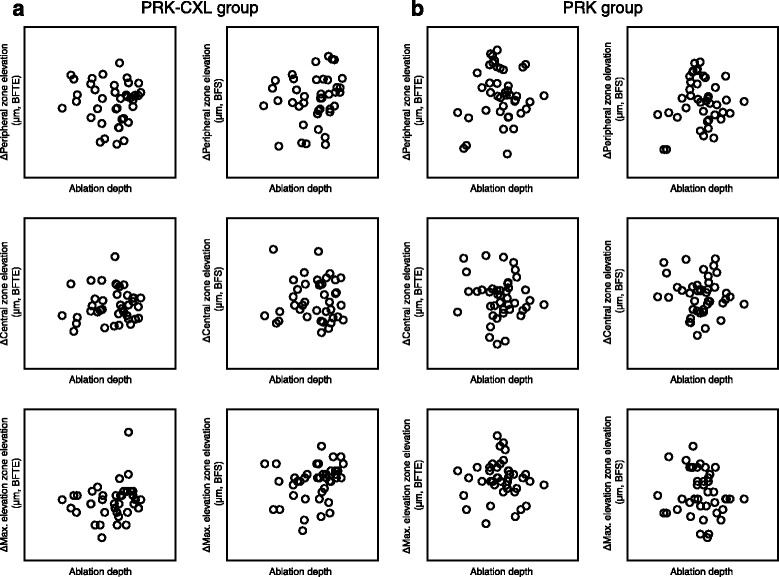
Correlations between changes in posterior corneal elevation values and ablation depth in eyes that underwent combined transepithelial photorefractive keratectomy and accelerated corneal collagen cross-linking and transepithelial photorefractive keratectomy alone. (**a**) PRK-CXL group, (**b**) PRK group. PRK-CXL = Combined transepithelial photorefractive keratectomy and accelerated corneal collagen cross-linking; PRK = transepithelial photorefractive keratectomy; Δ = change; Peripheral zone = measurements of 6-mm and 4-mm zone; BFTE = best-fit toric ellipsoid; BFS = best-fit sphere; Central zone = measurements of 2-mm zone and center; max. elevation = measurements of maximal elevation zone

In the PRK-CXL group, optical zone diameter (*r* = −0.314, *P* = 0.049) and spherical equivalent (*r* = −0.369, *P* = 0.019) were significantly correlated with the changes in posterior corneal elevation (BFS) at the maximal elevation zone. Additionally, a significant correlation was observed between total ablation zone diameter and changes in posterior corneal elevation (BFTE) at the peripheral zone (*r* = 0.336, *P* = 0.034). In the PRK group, significant correlations were observed between optical zone diameter and changes in posterior corneal elevation (BFS) at the maximal elevation zone (*r* = 0.339, *P* = 0.028) as well as between spherical equivalent and changes in posterior corneal elevation at the peripheral (BFTE: *r* = 0.349, *P* = 0.023; BFS: *r* = 0.322, *P* = 0.037) and maximal elevation (BFS: *r* = 0.393, *P* = 0.010) zones. There were no significant correlations between the changes in posterior corneal elevation at the peripheral, central, and maximal elevation zones and ablation depth in the PRK-CXL or PRK group (Fig. 2).

Upon multivariate linear regression analysis, none of the preoperative parameters were revealed to be the primary determinants of changes in posterior corneal elevation at the peripheral, central, and maximal elevation zones in either group.

## Discussion

In the present study, we investigated the changes in elevation of the anterior and posterior corneal surfaces after combined transepithelial PRK and accelerated corneal CXL and after transepithelial PRK alone. We demonstrated that combined transepithelial PRK and CXL induces significant elevation of the peripheral posterior corneal surface, but compared with transepithelial PRK alone, combined procedures show significant small amount of forward displacement of the peripheral posterior corneal surface. Furthermore, when analyzing posterior maximum corneal elevations, we found that combined procedures induce significant backward displacement of the posterior corneal surface.

Previous studies have reported forward displacement of the posterior corneal surface after myopic laser refractive surgery using scanning-slit topography and Scheimpflug tomography [1–5]. A recent study evaluating the change in posterior corneal elevation using swept-source optical coherence tomography after PRK described significant forward protrusion of the posterior corneal surface above BFS (8.0-mm-diameter), although magnitude was small (<4 μm) [22]. Detecting changes in the posterior corneal elevation after myopic laser refractive surgery is crucial because such changes are linked to postoperative keratectasia, a serious complication of refractive corneal surgery. The Scheimpflug tomography system used in this study enables measurements of changes in the posterior corneal elevation from the posterior corneal surface directly by elevation-based system.

In the present study, we hypothesized that application of prophylactic CXL while performing a laser ablation procedure could have a positive effect on regression of refractive effects and the forward or backward placement of the posterior corneal surface. We developed our hypothesis on the basis of recently published articles describing prophylactic CXL performed simultaneously with myopic LASIK surgery [17, 19]. The development of combined LASIK and CXL is based upon findings that myopia correction with LASIK in highly myopic eyes has shown a tendency to produce corneal steepening during long-term follow-up, consequently resulting in myopic shift. [23] Combined LASIK and CXL is thought to strengthen the cornea, especially in highly myopic eyes with thin residual stroma. Furthermore, clinical studies of the effects of combined LASIK and CXL in highly myopic eyes have demonstrated improved refractive and keratometric stability [19, 24, 25].

We analyzed posterior corneal elevations using elevation maps provided by Scheimpflug tomography. The Scheimpflug camera software calculates corneal elevation values by fitting a reference surface body, either a sphere (best-fit sphere; BFS) or an ellipsoid (best-fit toric ellipsoid; BFTE), to the corneal shape [26]. Previous studies have demonstrated the results of elevation data by fitting a sphere to the corneal shape, using the BFS as reference surface. In our study, however, in an attempt to improve the accuracy of posterior elevation measurements, we used the BFTE (8.0 mm in diameter) with a fixed eccentricity of 0.4 as the reference surface. Because the cornea is normally aspheric and ellipsoid, a toric ellipsoid approximates the actual shape of the normal cornea, thus demonstrating local elevation changes with more sensitivity than does the BFS [27, 28]. Sideroudi et al. reported that the results of posterior corneal elevations above the BFTE provide elevation measurements with the highest diagnostic capacity in suspected ectatic corneas [29].

Both groups in our study demonstrated peripheral anterior corneal elevations along with backward displacement of the central anterior corneal surface, regardless of the calculation method (BFS or BFTE). In cases with posterior corneal elevations, transepithelial PRK alone induced a significant forward displacement of the peripheral posterior corneal surface and a simultaneous backward displacement of the central posterior corneal surface. This finding is consistent with that of a previous publication that suggested a conceptual model for biomechanical central flattening as a direct consequence of severed corneal lamellae after myopic laser refractive surgery [30]. According to this theory, central lamellae are severed after surgery and are obliterated centrally; the remaining peripheral lamellae relax, and interlamellar distances expand with the reduction of lamellar tension [30]. This process allows the peripheral cornea to thicken, and ultimately an outward radial force in the peripheral cornea pulls laterally on the center and flattens the central cornea.

In line with changes induced by transepithelial PRK alone, combined transepithelial PRK and accelerated CXL induced a forward displacement of the peripheral posterior corneal surface and backward displacement of the central posterior corneal surface. However, the magnitudes of the changes were significantly smaller in combined transepithelial PRK and accelerated CXL than in transepithelial PRK alone when the BFTE was used as the reference surface. Furthermore, an analysis of posterior maximum corneal elevations demonstrated that combined transepithelial PRK and accelerated CXL induced a significant backward displacement of the posterior corneal surface.

We believe that because a prophylactic CXL intervention strengthens the corneal tissue, an outward radial expansion force in the periphery may cause little pull on the underlying intact corneal tissue. Accordingly, we found peripheral posterior corneal elevation and central corneal flattening that were small in magnitude in the combined PRK and accelerated CXL group. Additionally, backward displacement of the posterior corneal surface at maximum corneal elevations is expected to be attributed to the presence of a prophylactic CXL intervention.

CXL can provide biomechanical stability through corneal stiffening by causing formation of additional covalent connections between collagen fibers that consequently stabilize stromal collagen fibers and harden the structure of the collagen [11, 12]. In one study evaluating the effect of CXL on ectasia after excimer laser refractive surgery, keratocyte nuclei apoptosis in the anterior and intermediate corneal stroma caused significant tissue alteration during the first 3 months [31]. Stromal edema accompanied by keratocyte loss persists for 4 to 6 weeks and then gradually resolves with keratocyte repopulation and stromal collagen accumulation [32, 33]. Compared with conventional CXL, accelerated CXL is associated with a greater extent of keratocyte and corneal nerve apoptosis during the first 3 months following CXL [34, 35]. Based upon aforementioned changes in keratocyte apoptosis or repopulation, our future research will focus on investigating the relationship between posterior corneal elevations and changes in keratocyte nuclei after combined PRK and CXL during a longer follow-up period.

The present study had several limitations, including its retrospective design and relatively short period of observation. Another limitation is that we included two or one eyes in each patient. Additionally, this was not a randomized comparative study of contralateral eyes. In the present study, CCT of the PRK-CXL group was lower than that of the PRK group. Given that thinner corneas are prone to deformation, our results should be interpreted with caution. To validate our results, we intend to perform further prospective, randomized, contralateral paired-eye, clinical trials evaluating the effects of PRK with or without accelerated corneal CXL alone on posterior corneal elevations.

## Conclusions

A forward displacement of the peripheral posterior corneal surface was induced in both groups, along with a backward displacement of the central posterior corneal surface, regardless of the calculation method. Most notably, transepithelial PRK combined with a prophylactic CXL intervention significantly reduced the magnitudes of displacement of the peripheral and central posterior corneal surfaces, accompanied by the backward displacement of the posterior corneal surface at maximum corneal elevations when the BFTE was used as the reference surface.

## Abbreviations

BFS, best-fit sphere; BFTE, best-fit toric ellipsoid; CCT, central corneal thickness; CDVA, corrected distance visual acuity; CXL, collagen cross-linking; LASIK, laser in-situ keratomileusis; MRSE, manifest refraction spherical equivalent; PRK, photorefractive keratectomy; UDVA, uncorrected distance visual acuity
